# The Preparation of Electrolyte Hydrogels with the Water Solubilization of Polybenzoxazine

**DOI:** 10.3390/gels9100819

**Published:** 2023-10-14

**Authors:** Yutaka Ohsedo, Ami Kaneizumi

**Affiliations:** 1 Division of Engineering, Faculty of Engineering, Nara Women’s University, Kitauoyahigashi-machi, Nara 630-8506, Japan; 2 Graduate School of Human Centered Engineering, Nara Women’s University, Kitauoyahigashi-machi, Nara 630-8506, Japan

**Keywords:** polybenzoxazine, double network gel, hydrogels, hydrogel electrolyte

## Abstract

Polybenzoxazine (PBZ) exhibits excellent heat resistance, and PBZ derivatives have been designed and synthesized to achieve high performance. However, the application range of PBZ is limited by the strong interactions between molecular chains and its low solubility in organic solvents, thereby limiting its processability. This study focused on the benzoxazine structure as the molecular backbone of new hydrogel materials that can be applied as electrolyte materials and prepared functional gel materials. Here, we prepared hydrogels by water-solubilizing PBZ derivatives, which typically exhibit low solubility in organic solvents. Although studies on the hydrophilization of PBZ and its complexation with hydrophilic polymers have been conducted, no studies have been performed on the hydrogelation of PBZ. First, the phenol in the organic solvent-insoluble PBZ thin film obtained after the thermal ring-opening polymerization of the monomer was transformed into sodium phenoxide by immersion in a NaOH aqueous solution to water-solubilize it and obtain a hydrogel thin film. Although the hydrogel thin film exhibited low mechanical strength, a free-standing hydrogel film with improved strength was obtained through the double network gelation method with an acrylamide monomer system. The physical properties of the polymer composite hydrogel thin film were evaluated. The ionic conductivity of the hydrogel thin films was in the order of 10^−4^ S cm^−1^, indicating the potential of PBZ as an electrolyte hydrogel material. However, improving its ionic conductivity will be undertaken in future studies.

## 1. Introduction

Polybenzoxazine (PBZ) is obtained with the ring-opening polymerization of benzoxazine cyclic monomers. PBZ is classified as a phenolic resin; however, it is researched as a high-performance polymer that differs from phenolic resins owing to its unique properties [1,2,3,4,5,6]. Ring-opening polymerization is performed by heating a cyclic monomer, which can be easily obtained with reacting phenols, primary amines, and formaldehyde. The resulting PBZ resins exhibit remarkable heat resistance, flame resistance, surface properties, electrical and electronic properties, composite material properties (owing to the molecular and network structure of PBZ as a composite material), and optical properties. PBZ has attracted attention as a flame-retardant polymer material. An improvement in its performance is being sought with the addition of inorganic fillers, which have been confirmed to be flame-retardant [7]. PBZ has been demonstrated to exhibit a low surface-free energy comparable to that of polytetrafluoroethylene [8,9]. Synthetic research is underway to apply PBZ to coatings to obtain water-repellent surfaces with high performance and low surface-free energy [10,11]. The application of PBZ to coating materials on metal surfaces, etc., utilizing hydroxyl groups in PBZ, is also being investigated [12,13]. Regarding the electrical and electronic properties of PBZ, battery materials for cathode electrodes for Li–S systems obtained by introducing S into PBZ [14] and electrochromic materials obtained by introducing π-conjugated moieties such as triphenylamine moieties into PBZ [15,16] are undergoing investigation. Regarding composite materials, various property improvements or controls have been investigated, such as the control of thermal conductivity using composites [17], composites with carbon materials [18], and polymer materials [19], by harnessing the reactivity of the phenolic hydroxyl group of PBZ. In addition, as an example of research on adding optical properties to PBZ, PBZ with both photoluminescence and heat resistance has been reported [20].

A reason for these exceptional PBZ properties is the presence of intra- and intermolecular hydrogen bonds in the polymer chain [1]. This suggests the possibility of improving and controlling the physical properties by design synthesis and chemically modifying the molecular structure of the monomer. For example, the synthesis of high-molecular-weight PBZ by introducing a PBZ monomer into the main chain of azomethine polymers [21], the synthesis of heat-resistant PBZ resins from polymers in which PBZ monomers are bonded at the biphenyl moiety [22], the synthesis of PBZ with improved mechanical properties by introducing a polyrotaxane structure [23], the synthesis of nitrogen-enriched PBZ for supercapacitors [24], the introduction of reversible disulfide bonds [25], and the endowment of PBZ with self-healing properties through the dimerization of coumarins introduced into the side chains [26] have been investigated. Although molecular design has been conducted to improve the performance of the unique PBZ properties, studies are underway to obtain monomer raw materials from ecological and green resources. A green, high-performance PBZ resin from cardanol, an agro-waste phenol obtained from cashew nut shells, has been reported [27]. The research and development of green, high-performance PBZs from a sugar-based chemical, isosorbide [28], and cardanol [29] have been reported. In addition, an attempt to obtain PBZ from bio-based feedstock has been reported [30]. Thus, various molecularly designed amine and phenol derivatives can be utilized to adjust the required properties at the molecular structure level for specific applications. However, although PBZ monomers are soluble in organic solvents, PBZ resins generally exhibit low solubility in organic solvents. This low solubility has limited their application range. If PBZ can be solubilized, this would improve its moldability and expand its application possibilities to various material forms.

Polymer hydrogels [31,32], which have recently been expected to be applied as biomaterials [33,34], are conventionally regarded as brittle because of their low mechanical strength. However, efforts have been devoted to increasing their strength. The weakness of conventional gels has been attributed to various heterogeneities in the network structure [35,36], and research has been performed on high-strength gels. For example, gels obtained through the double network (DN) method, in which a rigid first network forming the gel structure and a second network compensating for the weakness of the first network are formed [37,38,39]; nanocomposite gels with long and uniform distances between cross-linking points using clay minerals as cross-linking agents [40,41]; polyrotaxane gels in which the rotaxane units function as movable cross-linking points similar to pulleys and efficiently distribute the force applied to the gel [42,43,44,45]; and highly uniform tetragels, which can be precisely controlled by combining tetrapod-type reactive monomers [46,47], are successful examples. In particular, gels obtained through the DN method exhibit a structure in which a hard and brittle electrolyte polymer (the first network) and a flexible and stretchable neutral polymer (the second network) are intermingled at the molecular level. With poly(2-acrylamido-2-propane sulfonic acid) gel as the first network and polyacrylamide gel as the second network, the synthesized gel demonstrates an extremely high strength that cannot be easily cut using a cutter. The mechanism of its high strength is being clarified, and high toughness is also being achieved [48]. These design guidelines for the high strength of hydrogels contribute to gels and the high strength of various polymeric materials [49].

**Figure 1:**
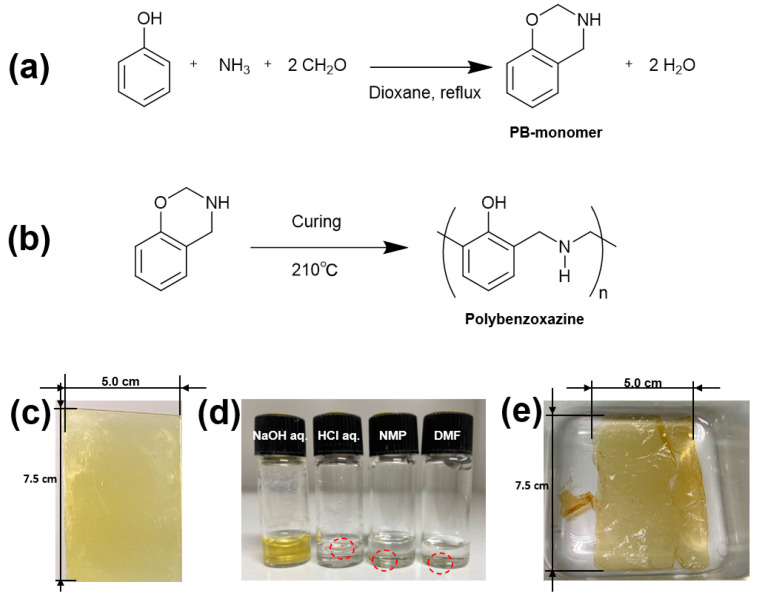
(**a**) Synthesis of PBZ monomer (PB-monomer); (**b**) synthesis of PBZ in this study. Photographs of the (**c**) PBZ thin film on a glass substrate (5.0 cm × 7.5 cm) obtained after curing PB-monomer thin film, (**d**) solubilization test of the PBZ thin film (the red circles indicate the insoluble PBZ thin film), and (**e**) NaOH-treated PBZ film (PB film).

As an investigation of the application of PBZ in functional materials, this study utilized PBZ in the hydrogel state through water solubilization. Several attempts, including the cationization of the main-chain nitrogen atom of PBZ with quaternary ammonium salts [50], the introduction of arbutin (a monosaccharide) and furfuryl groups into PBZ monomers [51], the formation of inclusion complexes of PBZ with cyclodextrins [52], the introduction of a furfuryl group into PBZ monomers [53], and cellulose grafting to PBZ [54], have been made to water-solubilize PBZ. Although studies have been performed on the synthesis of cross-linked PBZ using the amino group of the water-soluble polymer chitosan [55], no studies have been reported on the properties of PBZ as a hydrogel. Here, the water solubilization of PBZ was achieved with the sodium phenoxidation of the phenolic moiety by immersion in a NaOH aqueous solution to obtain a hydrogel. The DN method was employed to increase the strength of the hydrogel and expand the application range of the hydrogel thin film (Figure 1a). A water-soluble polymer, polyacrylamide, was selected as the second network. The mechanical and electrical properties of the obtained hydrogel thin film and the DN-enhanced hydrogel thin film were evaluated, and the application of the PBZ hydrogel thin film as a new hydrogel electrolyte membrane material was examined. Related to the present study, a study on the application of dry PBZ membranes with polyethylene oxide segments to solid polymer electrolytes is reported instead of the hydrogels of this study [56]. In relation to the research on the electrical properties of hydrogels, there is a review of research on the application of an electret to biomedical materials using hydrogel thin films [57].

## 2. Results and Discussion

A PBZ monomer, 3,4-dihydro-2H-1,3-benzoxazine (PB-monomer), was synthesized from aniline, ammonia, and formaldehyde according to a reported synthetic method [58] (Figure 1a) and obtained as a yellow-white solid. A 30 wt.% tetrahydrofuran solution of the synthesized PB-monomer was spin-coated onto a glass substrate at 1000 rpm for 30 s to form a monomer thin film, which was dried at 70 °C for 1 h. The ring-opening thermal polymerization (Figure 1b) of the resulting light-yellow PB-monomer thin film (10 µm thick) was performed by setting a hot stage to 210 °C and heating for 6 min. The examination of the weight change after heating revealed a weight loss of approximately 21.0%. Afterward, a dark-yellow polymer thin film was obtained (Figure 1d). The film was stuck on the glass substrate and could not be removed as a free-standing film. Therefore, the solubility of the film in acidic and alkaline aqueous solution systems and organic solvents was evaluated for samples in which the polymer thin film was scraped off in small pieces and soaked in a 1.0 M NaOH aqueous solution, a 1.0 M HCl aqueous solution, *N*-methyl-2-pyrrolidone (NMP), or *N*,*N*-dimethyl formamide (DMF) (Figure 1e). For a sample considered to have low molecular weight due to a short heating duration of 90 s at 210 °C, we examined its solubility at room temperature and at 100 °C. In NaOH aq., the film softened, and slight dissolution was observed. Based on these results, the polymer thin film on the glass substrate heated at 210 °C for 6 min was immersed in NaOH aq. in expectation that the film might peel off from the glass substrate. The polymer thin film could be peeled off from the glass substrate in the 1.0 M NaOH aq. while maintaining its thin-film state and was obtained as a free-standing polymer thin film. The polymer thin film was further immersed in pure water, and the solution was changed daily for approximately 4 days to purify the film, resulting in a free-standing polymer thin film (abbreviated as PB, Figure 1e). The weight of the polymer thin film after immersion in deionized pure water was compared with that after heat drying at 70 °C for 1 day, and a decrease in weight was observed after heat drying. The degree of swelling of the polymer thin film was evaluated using a weight standard comparing the swollen and dry states (Table 1). The thin film had q = 1.5 and was swollen with water. The polymer thin film obtained after the NaOH aq. treatment was observed to be a hydrogel capable of retaining water. The thicknesses of the PB hydrogel film and dry PB hydrogel film were 30 and 20 μm, respectively.

**Figure 2:**
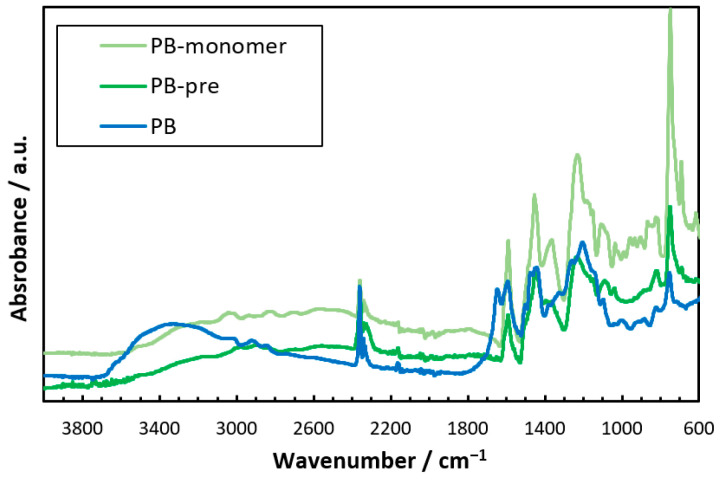
ATR-FTIR spectra of PB-monomer, PB-pre, and PB.

The dried hydrogel films of PB, pretreated PB (the PBZ film scraped off the glass substrate is denoted by PB-pre), and PB-monomer (Figure 2) were subjected to attenuated total reflectance Fourier-transform infrared spectroscopy (ATR-FTIR). The ATR-FTIR spectra showed that PB exhibited a broad absorption band between 3200 and 3700 cm^−1^. This was not observed for PB-monomer or PB-pre. This absorption band slightly shifted to an absorption band between 3500 and 3750 cm^−1^ derived from the OH stretching of the phenolic hydroxyl group [59,60]. This band was considered to be based on the –ONa generated by the neutralization reaction of the phenolic hydroxyl group in PB with NaOH upon water solubilization [61]. The results showed that the obtained hydrogel was a PBZ hydrogel thin film with –ONa as a water-soluble group. The mechanical strength of the PBZ hydrogel thin film was low, and the film could tear if mishandled during analysis. Thus, we endowed the PBZ thin film with mechanical strength using the DN method to increase its application range.

**Figure 3:**
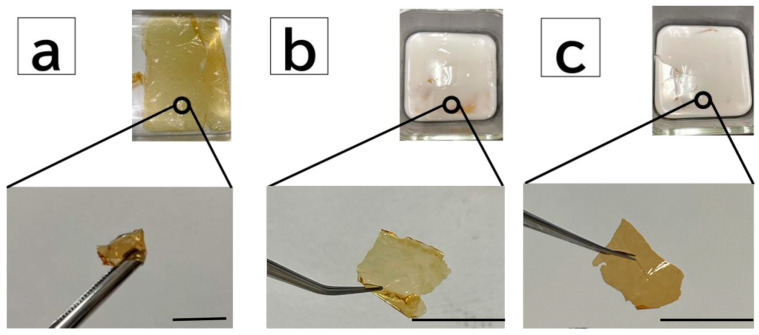
Images of DN PBZ gels: (**a**) PB film (bottom) after immersion in pure water (top). (**b**) PB-A1-10 film (bottom) removed from polymerized material (top) after employing the DN method. (**c**) PB-A1-5 film (bottom) removed from polymerized material (top) after employing the DN method. Scale bar: 1.0 cm.

To employ the DN method for the PBZ hydrogel thin film, polyacrylamide gel (acrylamide monomer (AAm) and cross-linker methylenebisacrylamide (BIS)) were formed as the second network. Hereafter, the poly AAm gel with a 1/10 molar ratio of BIS to 2.0 M AAm is referred to as “A1-10”, and that with a 1/5 molar ratio of BIS is denoted by “A1-5”. The PBZ hydrogel thin films are denoted by “PB”, and those with a 1/10 molar ratio of BIS to 2.0 M AAm using the DN method are denoted by “PB-A1-10”. Those with a 1/5 molar ratio of BIS to 2 M AAm are denoted by “PB-A1-5”. Figure 3 shows the samples after equilibrium swelling with pure water. The degree of swelling of each cross-linked sample is listed in Table 1. The degree of swelling represents the difference in cross-link density. A high degree of swelling indicated a low cross-link density, and vice versa. Therefore, the lower the cross-link density, the more the solution could be retained in the three-dimensional network of the gel. In AAm gels, A1-5, with a higher cross-linker concentration, had a higher degree of swelling than did A1-10, and A1-5 was considered to have a lower cross-link density than was A1-10 (q = A1-10 < A1-5; cross-linker density = A1-10 > A1-5). The swelling degree of PB was the highest, followed by those of PB-A1-10 and PB-A1-5. The order of PB-A1-10 and PB-A1-5 was the same as that of the AAm gel (q = PB < PB-A1-10 < PB-A1-5; cross-linker density = PB > PB-A1-10 > PB-A1-5).

**Figure 4:**
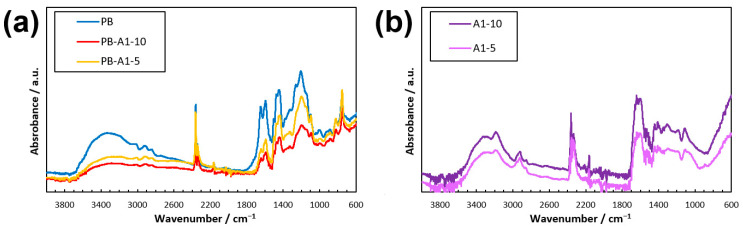
ATR-FTIR spectra of DN PBZ gels. (**a**) PB after DM method. (**b**) Polyacrylamides.

The dried samples of the DN PBZ hydrogel thin film were subjected to ATR-FTIR. Figure 4 shows that PB exhibited a gradual peak between 3200 and 3700 cm^−1^, which was not observed for PB-monomer and PB-pre. Similar peaks were observed for PB-A1-10 and PB-A1-5, as shown in Figure 4, although they were not as prominent as those for PB. They slightly shifted to between 3500 and 3750 cm^−1^, which may have been due to a change in the PBZ structure from –OH to –ONa upon water solubilization. We believe that the peak of the AAm gel between 3100 and 3400 cm^−1^ shown in Figure 4 differs from the 3200–3700 cm^−1^ peaks of PB, PB-A1-10, and PB-A1-5.

**Figure 5:**
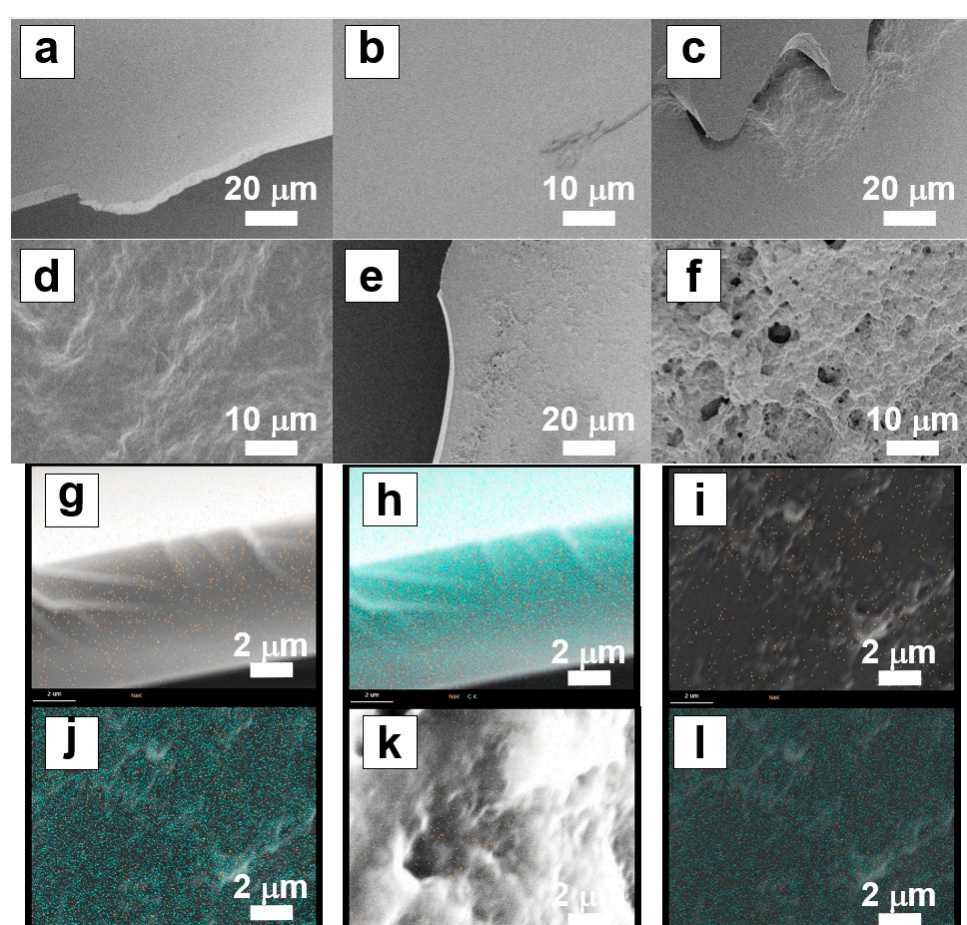
SEM and EDS images of PBZ xerogels. SEM images: (**a**,**b**) PB, (**c**,**d**) PB-A1-10, and (**e**,**f**) PB-A1-5. EDS images (orange dot: sodium atom, green dot: carbon atom): (**g**,**h**) PB, (**i**,**j**) PB-A1-10, and (**k**,**l**) PB-A1-5.

The scanning electron microscopy images of the surface morphology of the freeze-dried samples of the PBZ hydrogel films are shown in Figure 5. The PB surface did not feature a characteristic morphology; however, the PB-A1-10 and PB-A1-5 surfaces exhibited wavy and porous morphologies, respectively. The reason for this difference in surface structure in the different gels is unclear; however, it is thought that these shapes are related to DN formation. The elemental mapping of PBZ with energy-dispersive X-ray spectroscopy revealed the presence of 1–2% Na atoms, suggesting that the PBZ hydrogel film can be converted into –ONa by the NaOH solution. However, since the presence of Na atoms was observed at the surface, it is presumed that this does not signify the concentration of Na atoms in the bulk of the thin film.

**Figure 6:**
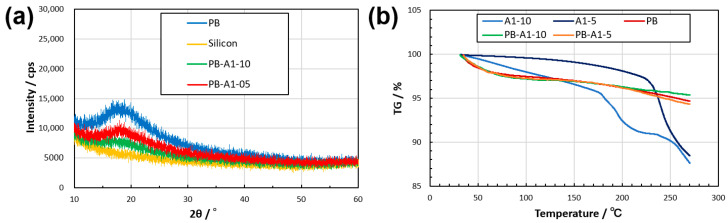
XRD (**a**) and TGA (**b**) results for the dried thin films of PBZ. The results of XRD include a silicon nonreflective plate as a substrate.

To evaluate the polymer aggregation structure in the thin film, the dried samples were subjected to X-ray diffraction (Figure 6a). This may imply that the polymers in the films did not contain significant crystalline regions and were mainly in an amorphous state. The thermogravimetric analysis (TGA) of the thermophysical properties of the dried thin films showed that PB, PB-A1-10, and PB-A1-5 remained at approximately 95% TG, even with an increase in temperature. Furthermore, a comparison of weight loss at 270 °C showed that PB-A1-10 exhibited a 1% reduction in weight loss over PB-A1-5 (Figure 6b), suggesting that a trend exists for DN-enhanced thermophysical properties of PBZ.

**Figure 7:**
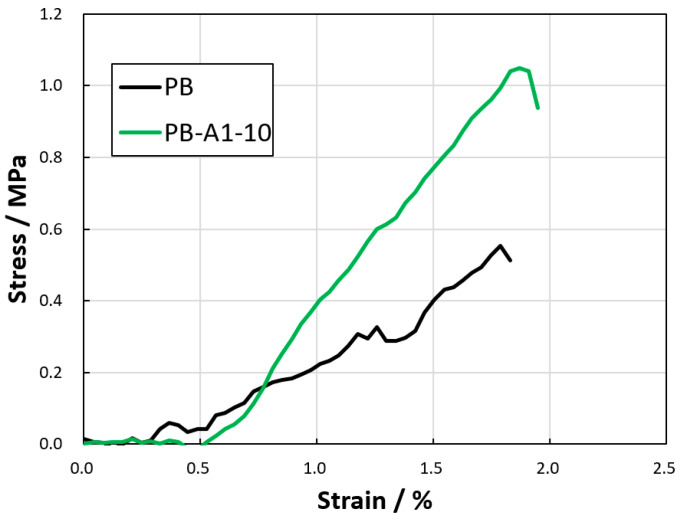
Tensile tests of DN PBZ hydrogels.

To evaluate the degree of improvement in the mechanical strength of the thin films due to the DN method, tensile tests were performed on the thin-film samples cut into strips. Considering that the PB-A1-5 film broke when it was removed from the acrylamide gel (the second network) and the samples decreased in size, tensile tests were performed on the PB and PB-A1-10 gel membranes only. Figure 7 shows the stress–strain curves of the PB hydrogels. The breaking stresses of PB and PB-A1-10 were 0.55 and 1.05 MPa, respectively. The stress–strain of PB-A1-10 also exceeded that of PB. Thus, the DN method improved the strength of the PBZ hydrogel film. However, even with the DN method, the PBZ hydrogel was not so strong that it could not be cut with a cutter, as has been reported for DN gels [37,38,39], probably because the molecular weight between the cross-linking points of the PB gel was not sufficiently high to obtain a high-strength gel.

Finally, the ionic conductivity of the PBZ hydrogel films was evaluated at 25 °C (Table 2) to be approximately 10^−4^ S cm^−1^. Na^+^ was considered to be the charge carrier for this ionic conduction since the thin films were hydrogelated by the sodiation of the phenolic hydroxyl groups. Compared with the ionic conductivities of typical electrolyte polymers—Nafion (10^−2^ S cm^−1^ [62]), polyether/Li salt-type solid polymer electrolytes (10^−3^ to 10^−5^ S cm^−1^ [63]), and gel-like polyelectrolytes containing electrolyte solution (10^−3^ S cm^−1^ [64])—those of the dry and gel membranes of PB, PB-A1-10, and PB-A1-5 were comparable to those of ether-based polyelectrolytes but were not as high as those of Nafion and gel polyelectrolytes. This may be because Na^+^, which is considered a carrier, easily dissociates in the gel state. The low ionic conductivity may have been due to the low concentration of Na^+^ carriers; however, further evaluation of the Na concentration is required. The ionic conductivity of the PBZ hydrogel thin film was evaluated by changing the temperature in 10 °C increments from 5 °C to 45 °C. Within this temperature range, there was practically no temperature dependence on ionic conductivity.

## 3. Conclusions

PBZ, known for its insolubility, was water-solubilized with treatment in an aqueous NaOH solution. The phenolic hydroxyl group of PBZ was sodiated to sodium salt in order to synthesize PBZ hydrogel thin films, the first material form of PBZ. The obtained hydrogel thin film exhibited low mechanical strength; therefore, the DN method for increasing the strength of hydrogels was employed, using the PBZ hydrogel thin film as the first network and acrylamide as the second network. In addition, the ionic conductivities of the PBZ hydrogel thin film and DN membrane were comparable to those of ether-based polyelectrolytes. The ionic species that contributed to this ionic conductivity were thought to be Na^+^. However, the ionic conductivities were not comparable to those of Nafion or gel polymer electrolytes, and an improvement in the ionic conductivity is required. This study demonstrates the potential of PBZ hydrogels as ion-conducting electrolyte gels and shows the possibilities of new molecularly structured electrolytes.

## 4. Materials and Methods

Acrylamide (AAm) was recrystallized with *n*-hexane, *N*,*N*′-methylenebisacrylamide (BIS) was recrystallized from methanol, and 2,2′-azobis(2-methylpropionamidine) dihydrochloride (ABMPA) was used as obtained (these reagents were purchased from Tokyo Chemical Industry Co., Tokyo, Japan). Other reagents, solvents, and ammonia water (25%) were purchased (Wako Pure Chemical Industries, Ltd., Tokyo, Japan) and used as received. Pure water was produced with an Elix UV 3 Milli-Q integral water purification system (Nihon Millipore K.K., Tokyo, Japan).

PBZ monomer, 3,4-dihydro-2H-1,3-benzoxazine (PB-monomer), was synthesized according to the method described in the literature [58]. Briefly, 86.5 mL of formaldehyde (0.40 mol) and 120 mL of dioxane were mixed in a 500 mL four-neck and stirred to keep the solution temperature below 10 °C; a 30 mL dioxane solution of 26.4 mL of ammonia water (0.50 mol) was dropped into the flask in 5 min. Then, to this mixture, a 100 mL dioxane solution of 47.5 g of phenol (0.50 mol) was added dropwise in 5 min, followed by refluxing at 110 °C for 7 h in an oil bath. The reaction solution was then allowed to cool to room temperature, placed in diethyl ether, washed three times with aqueous NaOH solution, washed once with pure water, and dried over Na_2_SO_4_; the solvent was removed under reduced pressure and vacuum heat-dried to afford the yellow solid, PB-monomer (27.7% yield on NH_3_ basis).

The molecular structure of the synthesized PB-monomer was determined with ^1^H-NMR (JNM-ECZ400, JEOL Ltd., Tokyo, Japan, DMSO-*d_6_*, 400 MHz), ^13^C-NMR (JNM-ECA600, JEOL Ltd., 150.9 MHz), and high-resolution mass spectrometry (HRMS, Matrix-Assisted Laser Desorption/Ionization Time of Flight Mass Spectrometer JMS-S3000, JEOL Ltd. Japan, matrix: α-cyano-4-hydroxycinnamic acid), respectively.

PBZ monomer: 1H-NMR (DMSO-*d_6_*, δ in ppm) 10.18 (s, 1H, -NH-), 7.21–6.75 (m, 4H, aromatic CH), 4.88–3.28 (m, 4H, -CH_2_-). 13C NMR (DMSO-*d_6_*, δ in ppm) 157.37, 129.46, 128.35, 123.15, 118.87, 115.27, 66.41, 52.9. HRMS: calcd for C_8_H_9_NO, 135.07; found m/z = 136.08 [M+H]^+^.

Spin-coated films were prepared using an Opticoat SpinCoater MS-A150 (MIKASA CO., LTD, Tokyo, Japan) under the conditions described in the main text.

The thermal polymerization of the spin-coated films was performed using an RSH-1D hot stirrer (AS ONE CORPORATION, Osaka, Japan) with a thermocouple set.

Thin film production with the DN method was performed as follows. To form a second network of polyacrylamide in the water-soluble thin film obtained with the method described in the text, two aqueous solutions of water-soluble monomer: AAm, cross-linking agent: BIS, and initiator ABMPA (concentrations, AAm: 2.0 M, BIS: 0.2 M, ABMPA 2.0 mM, AAm: 2.0 M, BIS: 0.2 M, ABMPA BIS: 0.4 M, ABMPA 2.0 mM, 5 mL of aqueous solution, respectively) were placed in a refrigerator for 2 days. Nitrogen bubbling was performed twice for a total of 20 min each, immediately after immersion of the water-solubilized polymer films and immediately before thermal polymerization. Thermal polymerization was then carried out in a water bath at 60 °C for 8 h; the resulting gel was immersed in pure water to achieve equilibrium swelling, and the thin films were removed from the AAm gel. To remove unreacted monomers, the pure water was replaced every day for 4 days.

Swelling measurements of samples (PB, PB-A1-10, and PB-A1-5) were performed using the weight of the sample after wiping the water from the surface of the sample and the weight of the sample after heating and drying at 70 °C for 1 day. The formula, q = (swollen weight)/(dry weight), was used to calculate the degree of swelling q.

The attenuated total reflectance Fourier-transform infrared spectroscopy (ATR-FTIR) evaluation was performed using an FTIR6600 spectrometer (JASCO Corporation, Tokyo, Japan) equipped with a single-bounce diamond attenuated total reflectance unit.

Scanning electron microscopy (SEM) was performed using an SU-8020 TYPE II EDS (Hitachi High-Tech Corporation, Tokyo, Japan) and the energy-dispersive X-ray spectroscopy (EDS) analysis was performed using a Genesis APEX2 Apollo XL (AMETEK, Tokyo, Japan); measurements were performed on samples of sputtered Pt films (thickness: approx. 10 nm) deposited on vacuum-heated dried samples using the E-1045 multifilm processor (Hitachi High-Tech Corporation, Tokyo, Japan).

X-ray diffraction (XRD) data were measured on a silicon nonreflective plate at 25 °C in a fully automated multipurpose X-ray diffractometer SmartLab (Rigaku Corporation, Tokyo, Japan).

Thermogravimetric analysis (TGA) data were measured on an STA7300 (Hitachi High-Tech Corporation, Tokyo, Japan) at 10 °C/min under nitrogen flow using an aluminum sample container.

The tensile testing of the membranes was performed on a tabletop universal testing machine MCT-2150 (A&D Company, Limited, Tokyo, Japan). The membrane was cut into 5 mm × 15 mm tapes, set so that the length of the tape sample between the fixtures was 5 mm, and the relationship between stress and strain was recorded when the tape was pulled at 10 mm/min.

For the ion conductivity measurement, a film sample was placed on a 0.5 mm thick aluminum plate so that the measurement area was 5.0 mm × 5.0 mm, fixed with a clip, and placed in an electronically cooled constant-temperature chamber TB-1 (BAS Inc., Tokyo, Japan) set at 25 °C. The sample was measured using an LCR meter IM3536 with an L2000 probe (HIOKI E.E. CORPORATION, Nagano, Japan). The impedance and phase angle were measured at an applied voltage of 1.0 V and a frequency of 5 to 5 MHz, and a Cole–Cole plot was created and calculated considering the film thickness described above.

## Figures and Tables

**Table 1:** Degree of swelling of thin hydrogel film samples.

Sample	q
PB	1.5
PB-A1-10	1.5
PB-A1-5	1.9
A1-10	4.9
A1-5	5.9

**Table 2:** Ionic conductivities of the thin hydrogel film samples.

Sample	Ionic Conductivity (S cm^−1^)
Dried PB	6.7 × 10^−5^
PB	1.9 × 10^−4^
PB-A1-10	8.1 × 10^−5^
Dried PB-A1-10	8.9 × 10^−5^
PB-A1-5	1.3 × 10^−4^
Dried PB-A1-5	5.8 × 10^−5^

## Data Availability

Not applicable.
